# Impact of laboratory involvement in the characterization of B12 hypervitaminosis in clinical practice

**DOI:** 10.1515/almed-2024-0098

**Published:** 2024-08-14

**Authors:** Sara Fernández-Landázuri, Ramón Baeza-Trinidad, Iván Bernardo González

**Affiliations:** 118003 Department of Biomedical Diagnostics, Hospital San Pedro, Logroño, Spain; Service of Internal Medicine, Hospital San Pedro, Logroño, Spain

**Keywords:** medical consultations, costs, B12 hypervitaminosis, interference, macro-B12

## Abstract

**Objectives:**

Unexplained B12 hypervitaminosis (HB12) in asymptomatic patients leads to a cascade of medical consultations and diagnostic tests aimed at determining its etiology. The objective of this study was to assess the efficacy of the laboratory getting involved in the detection and elimination of immune complexes with vitamin B12 in clinical practice and its economic impact.

**Methods:**

A retrospective longitudinal study was undertaken to assess the laboratory strategy of detecting B12 macrovitamin (macro-B12) in patients with HB12 >1,000 pg/mL. The clinical characteristics of patients with HB12 referred to Internal Medicine (IM) in the pre- and post-implantation period of the new strategy were compared. Additionally, the healthcare costs of one-year follow-up were estimated.

**Results:**

The prevalences of HB12 in the pre- and post-implantation period were 3.9 % and 3 %, respectively. Macro-B12 explained 25 % of the HB12 cases initially detected. A 41 % reduction was observed in the number of patients with HB12 after the implantation of the new strategy, thereby resulting in a cost reduction of 5,000 €.

**Conclusions:**

The laboratory intervention for the detection of macro-B12 provides clear economic and clinical benefits in clinical practice.

## Introduction

Vitamin B12 (VB12), also known as cobalamin, is one of the most frequently measured analytes in clinical practice. This test is generally focused on VB12 deficiency, more specifically, on megaloblastic anemia and neuropsychiatric disorders associated with demyelination of the spinal cord, peripheral nerves and the brain [1], [2], [3]. However, there is an interest growing in B12 hypervitaminosis (HB12), which has long been overlooked and underestimated [4, 5].

A balanced diet helps meet nutritional needs for VB12, as human cells cannot synthetise this water-soluble vitamin. Cobalamin, the only vitamin exclusively synthetized by microorganisms, refers to different forms of vitamin B12, being adenosylcobalamin and methylcobalamin its biological forms, and cyanocobalamin and hydroxycobalamin the synthetic forms. This essential micronutrient is primarily found in food from animals like meat, dairy and eggs. It is also found in VB12-fortified foods and in an arsenal of oral B-vitamin supplements available over the counter. Cobalamin deficiency may require parenteral administration [2].

HB12 is defined as levels of VB12 above the upper limit of reference, which varies according to the analytical method used. HB12 has been associated with a wide variety of conditions, primarily involving the liver, the kidneys, the autoimmmune system and neoplasms [6]. There are multiple molecular mechanisms that mediate VB12 elevation, including the elevation of transporter proteins in neoplasms, massive release from hepatic reservoirs, decreased transcobalamin 2 production in liver disease or reduced transcobalamin 2 filtration in kidney disease [7]. There is an extensive literature about the association of HB12 with solid and hematologic neoplasms. Of the latter, chronic myeloid leukemia (CML), polycythemia vera (PV) and hypereosinophilic syndrome are of special interest. Indeed, VB12 measurement has been included as a minor and differentiation criterion for PV [6, 8], [9], [10]. In relation to solid neoplasms, HB12 has been associated with liver cancer and higher short-term mortality rates [11], [12], [13], [14], [15]. Hence, HB12 has been related to cancer and the associated mortality. However, there is no solid evidence supporting a causal relationship between HB12 and/or pharmacological treatment with VB12 and cancer [14].

In clinical practice, VB12 is measured by immunochemiluminescent assays. These assays are not exempt from analytical interferences, which lead to the misinterpretation of results and the suboptimal management of patients [16]. One of the main analytical interferences in VB12 determination is the formation of immune complexes of IgG or IgM immunoglobulins bound to circulating cobalamin, commonly referred to as macrovitamin B12 (macro-B12). This immune complex, which is functionally inactive, elevates VB12 concentration falsely and might even mask VB12 deficiency. The prevalence of macro-B12 interference is high, accounting for 10–30 % of HB12 [5, 17], [18], [19]; therefore, it is critical that laboratories be aware of this interference and adopt strategies to identify and eliminate it as much as possible [17, 20, 21].

Laboratories are increasingly aware of this analytical interference in the determination of VB12; however, no studies have been undertaken to assess the role of the laboratory in avoiding this interference and its relevance in clinical practice.

This study was conducted to assess the clinical and economic impact of appropriate HB12 characterization in the laboratory following the elimination of macro-B12 in patients with HB12 referred to the Unit of Internal Medicine of our hospital.

## Materials and methods

### Study design

**Figure 1:**
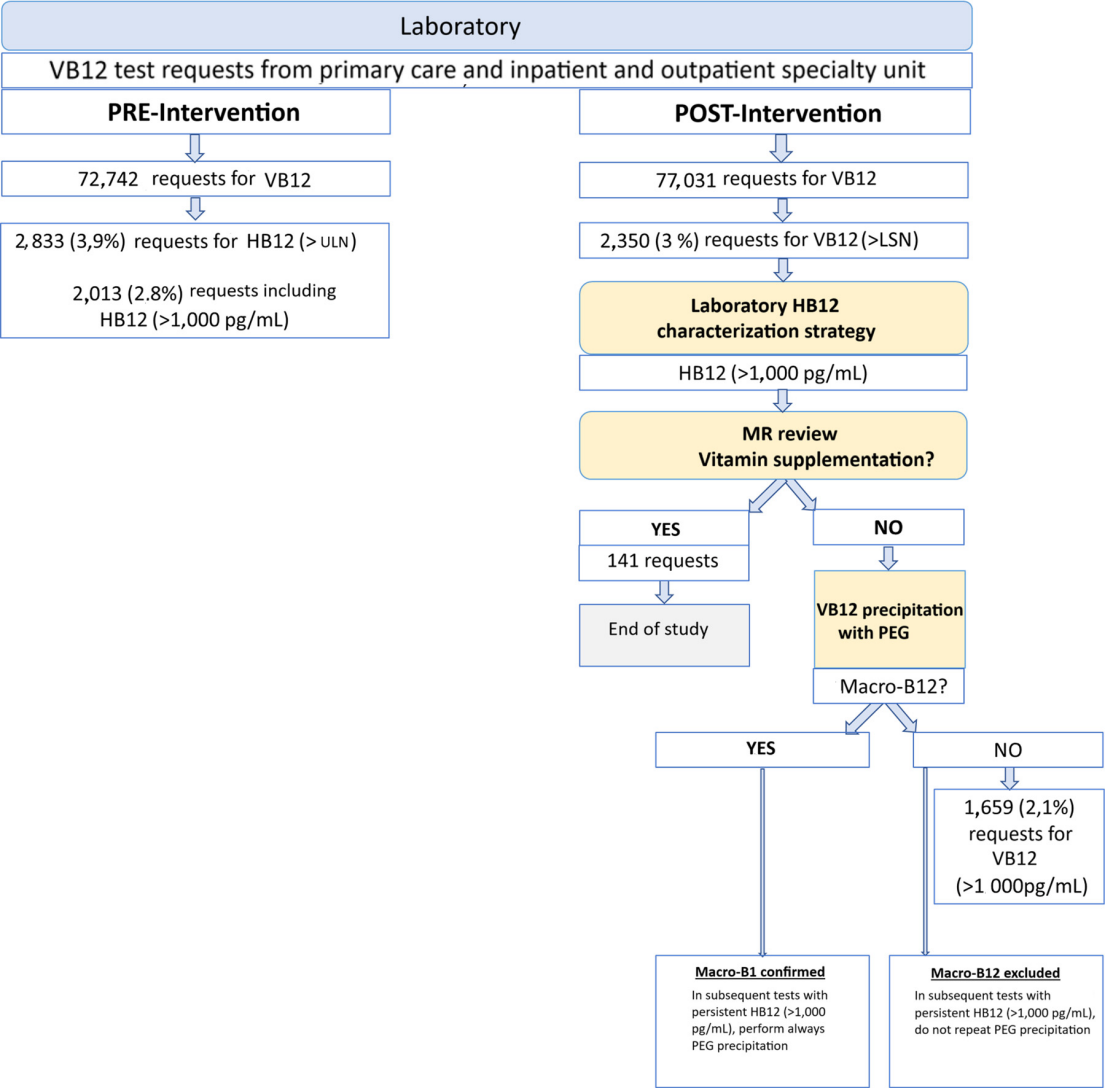
Algorithm for elevated B12. HB12: hypervitaminosis B12; MR: medical history LSN: upper limit of normal (197 to 771 pg/mL or 145-569 pmol/L); PEG: polyethylene glycol; VB12: vitamin B12.

A retrospective longitudinal study was carried out to assess the impact of macro-B12 detection by the laboratory on clinical practice and healthcare costs (Figure 1).

The study was approved by the Institutional Review Board (Ref CEImLAR P.I. 606).

### Characteristics of the laboratory and hospital

The laboratory of the Service of Laboratory Medicine is located in San Pedro University Hospital (HUSP, La Rioja, Spain), serving a population of 320,000 inhabitants. The laboratory receives samples from hospitalized patients, outpatient specialty consultations and primary care.

### Management of B12 hypervitaminosis

Diagnosis of patients with idiopathic HB12 referred to IM was established on the basis of a clinical evaluation, a complete biochemistry test including tumor markers and a proteinogram, an abdominal ultrasound, and a chest radiograph. If necessary, the study was complemented with a positron emission tomography-computed tomography (PET-CT).

### Laboratory intervention

Although the reference interval established in the literature for HB12 ranges from 197 to 771 pg/mL, HB12 was established in the presence of a VB12 concentration above 1,000 pg/mL. In the new strategy adopted by the laboratory to detect macro-B12, macro-B12 test results were automatically recorded in the Laboratory Information System (LIS) for patients with serum VB12 values >1,000 pg/mL.

Then, the head of the section reviewed the medical record of these patients for prescriptions of B12 supplementation in order to exclude patients receiving B12 supplementation.

Once vitamin supplementation was ruled out as the cause of VB12 elevation, we investigated potential analytical interference from macro-B12 through precipitation with polyethylene glycol (PEG).

When macro-B12 was detected and HB12 persisted in subsequent laboratory tests, precipitation with PEG was repeated. When the presence of macro-B12 was excluded in patients with persistent HB12, precipitation was not repeated.

### Laboratory method

Serum VB12 was quantified by competitive electrochemiluminescence immunoassay (ECLIA) in a Cobas 8000 (Roche Diagnostics, Mannheim, Germany) analyzer. The interval of reference reported by the manufacturer was 197–771 pg/mL.

VB12 immune complexes were detected by PEG precipitation. After precipitation, VB12 was determined in the supernatant. The percent recovery after correction for dilution effects ([s-B12 concentration after PEG precipitation x2/s-B12 concentration before PEG precipitation]×100) was automatically calculated in the LIS. The presence of macro-B12 was confirmed when the percent recovery was <60 %. The associated result was provided in the laboratory report including a comment indicating the estimated VB12 result in presence of macro-B12.

### Patients

The study sample included patients with HB12 (>1,000 pg/mL) seen in the IM consultation of the HUSP (the specialty consultation where this entity is studied) the year before (pre-intervention) and after (post-intervention) the strategy for the detection of macro-B12 was implemented in the laboratory.

The date of patient inclusion was the date of the first laboratory result of HB12 (pre- or post-intervention). One-year follow-up was performed since patient inclusion in the study. Exclusion criteria were loss to follow-up by IM and management of HB12 in other medical units.

The data collected included demographic (age and sex) and clinical data (presence of hypertension, dyslipidemia, hypothyroidism, hyperparathyroidism, anemia, cholelithiasis, heart failure, chronic respiratory disease, chronic kidney disease, liver disease, autoimmune diseases, hemochromatosis; cancer (hepatic, breast, colorectal, gastric, pancreatic and others); myeloproliferative, myelodysplastic, lymphoproliferative and metastatic syndrome). Other data was included as pharmacological (treatment with proton pump inhibitors and vitamin supplementation), morbimortality (number of hospitalized days and one-year mortality), analytical and healthcare data (number of consultations with IM, request for new laboratory tests, abdominal ultrasound, chest X-ray and other imaging tests).

The costs generated per patient were calculated from the billing rates for healthcare services reported by RiojaSalud, the public healthcare system of La Rioja, a northern region of Spain. The cost of detecting macro-B12 was calculated [cost of identification of patients with macrovitaminosis-B12 (€)=(No. of additional B12 tests*cost of B12 tests)/No. of patients identified]. The cost of vitamin B12 testing in our center was 1.29€. PEG, consumables, staff and intangible and other indirect costs were excluded from estimations.

### Statistical analysis

All statistical analyses were performed with the Statistical Package for the Social Sciences 22.0 (SPSS Inc., Chicago, IL, USA).

Non-Gaussian distribution was assessed by the Kolmogorov–Smirnov test of normality. Continuous variables were expressed as mean±standard deviation (SD) or as median and interquartile range (based on normality). Categorical data were presented as absolute numbers (n) and as percentages (%).

Chi-square test was used to assess differences in categorical variables across groups. Student’s t-test or Mann Whitney U test were used to compare unpaired samples, where appropriate. A bilateral p-value <0.05 was considered statistically significant.

## Results

During the study period, a 5.9 % increase in laboratory activity based on the number of VB12 requests received was observed. Additionally, in our Service of Internal Medicine, clinical activity increased by 4.6 %, according to the total number of consultations recorded (Figure 1).

In our healthcare area, the prevalence of HB12, defined as >1,000 pg/mL, was 3.9 % and 3 % in the pre- and post-intervention period, respectively.

In the post-intervention period, the 6 % of initial B12 elevations were due to B12 supplementation, with a prevalence of macro-B12 of 24.9 %. Ten cases of VB12 deficiency (<200 pg/mL) were incidentally identified by PEG precipitation, after VB12 values had been consistent with HB12.

The number of cases of HB12 managed in IM decreased considerable in the post-intervention period. A total of 61 patients with HB12 (7.2 % from the total consultations with IM) were seen in the pre-intervention period vs. 36 (4 %) in the post-intervention period, which accounts for a 41 % reduction. The population seen in IM in the pre- and post-intervention period had similar characteristics (Table 1). There were no statistically significant differences in terms of morbidity, except for the presence of diabetes mellitus (pre-intervention 22 % vs. post-intervention 6 %; p: 0.030) and anemia (pre-intervention 11 % and post-intervention 0 %; p: 0.034). As mentioned above, the new diagnostic approach to HB12 of unknown etiology involves a cascade of visits and additional complementary studies, standing out a decrease in the number of laboratory test requests and an increase in imaging study requests (Table 2).

**Table 1:** Clinical characteristics and morbimortality of patients with elevated levels of vitamin B12 referred to the Unit of Internal Medicine.

Characteristics	Pre-intervention (61 patients)	Post-intervention (36 patients)	p-Value
Age, years (mean, SD)	68±14	68±17	0.692
Sex, n; % women	39; 64 %	21; 58 %	0.368
Ethnicity, n; % Caucasian	58; 95 %	33; 92 %	0.160
Solid tumor, n; %	11; 18 %	3; 8.3 %	0.155
Hematologic tumor, n; %	5; 8.2 %	7; 19.4 %	0.970
Solid and hematologic tumor, n; %	16; 22.2 %	10; 27.8 %	0.454
Metastasis, n; %	2; 3.3 %	3; 8.3 %	0.454
1-year mortality, n, %	11; 18 %	10; 27.8 %	0.191

SD, standard deviation, %, percentage.

**Table 2:** Requests for laboratory tests and imaging studies for patients with vitamin B12 elevation referred to the Unit of Internal Medicine.

Complementary studies	Pre-intervention (61 patients)	Post-intervention (36 patients)	p-Value
Request for a repeat laboratory study after vitamin B12 elevation, n; %	48; 78.3	23; 63.9	0.970
Request for abdominal ultrasound after vitamin B12 elevation, n; %	14; 23.3	4; 11.1	0.110
Request for chest X-ray after vitamin B12 elevation, n; %	12; 20	6; 16.7	0.452
Request for PET-CT after vitamin B12 elevation, n; %	1; 1.7	36; 8.3	0.147

The cases of HB12 managed in IM consultations generated costs related to healthcare services and relevant complementary studies (Table 3). A reduction was observed in all costs in the post-intervention period, with a notable reduction of the cost of imaging tests, except for PET-CT (Table 3). Considering that the new diagnostic strategy based on VB12 testing by PEG precipitation required an investment of €2,433 in the laboratory, added to a difference of €7,528 in the total cost of patients seen in IM between the two periods, a total cost reduction of €5,095 was achieved. Standardization of the cost per patient was greater in the post-intervention period in relation to the costs of medical consultations and imaging tests (Table 3).

**Table 3:** Total and standardized cost per patient for healthcare services provided to patients with vitamin B12 elevation seen in the Unit of Internal Medicine.

Cost category	Pre-intervention (61 patients)	Post-intervention (36 patients)	Cost difference	Cost/patient pre-intervention	Cost/patient post-intervention	Cost/patient difference
Consultation IM, €	22,350	14,031	−8,319	366.4	389.8	+23.4
Lab analysis, €	1,537	782	−755	25.2	21.7	−3.5
Abdominal US, €	826	236	−590	13.5	6.6	−6.9
Chest X-ray, €	240	120	−120	3.9	2.2	−0.6
PET-CT, €	1,128	3,384	+2,256	18.5	94.0	+75.5
Total cost of healthcare services	26,081	18,553	−7,528	427.6	515.4	+87.8
Cost of laboratory services (HB12 characterization)	0	2,433	+2,433			
Total cost of IM strategy			−5,095			

IM, internal medicine; HB12, hypervitaminosis B12; €, Euro; US, ultrasound.

## Discussion

HB12 has been associated with a wide range of diseases, especially with liver disease, neoplasm and autoimmune diseases. Additionally, HB12 has been suggested as a prognostic marker o these diseases [6, 11, 12, 15]. The incidental finding of HB12 of unknown etiology in asymptomatic patients leads to a cascade of clinical consultations and complementary diagnostic tests to screen for potentially undiagnosed diseases [4, 7].

In this context, it is estimated that 60–70 % of medical decisions are based on laboratory results. Therefore, it is crucial that the laboratory provides reliable data to avoid generating erroneous results that lead to categorize healthy subjects as unhealthy, with the corresponding personal, occupational and economic implications [22], [23], [24], [25]. The occurrence of laboratory errors has decreased notably with the advent of new technologies, the standardization of analytical methods, and the implementation of quality control programs [24].

However, the methods used by clinical laboratories for testing VB12 are based on immunoassays, which are not free from analytical interferences [16]. The formation of immune complexes constitutes a relevant source of interferences. The reason is that when antibodies bind analytes *in vivo*, the characteristics of analytes change and analytes retain their capacity to react with immunoassays, thereby causing falsely elevated values. This interference has been extensively described and associated to prolactin. Hence, laboratories have implanted procedures for the detection and elimination of macroprolactin by PEG precipitation [25], [26], [27]. Other hormones, like TSH and FSH, added to some enzymes like creatine kinase, have also been suggested to cause this kind of interference [16, 28, 29]. There is growing interest from laboratories in detecting analytical interferences in VB12 tests, especially in asymptomatic patients with HB12 of unknown etiology. The gold standard method for detecting immune complexes is molecular exclusion chromatography. However, its inherent complexity and long turnaround time make it ineffective in clinical practice, as it occurs with gel-filtration chromatography [17, 30]. The clinical laboratory needs to implant feasible techniques and protocols such as PEG precipitation, which is? the method commonly used to detect macro-B12 in daily practice. In aqueous solution, this nonionic polymer is capable of precipitating high molecular weight proteins, such as immune complexes, in a simple, fast and economical way. Despite the nonspecific carry-over of other small molecules such as cobalamin, PEG precipitation is a convenient method for detecting macro-B12 and establishing diagnosis of HB12. Indeed, the prevalence of macro-B12 in our population is 24.9 %, which is consistent with the results reported for other cohorts of patients [17, 20, 21]. This high incidence warrants the implementation of protocols for avoiding this analytical interference by using a similar approach to the one used for macropolactin [27]. For this reason, despite its limitations, it is useful to include VB12 concentration after PEG precipitation and after correction for dilution effects on laboratory reports.

In general terms, the most frequent cause of HB12 is the administration of oral or intramuscular hydroxycobalamin or cyanocobalamin supplements [2, 7]. In our population, however, the prevalence of supplementation is lower than the prevalence of macro-B12 detected. This may be due to the fact that most of the vitamin supplements administered orally are not recorded in medical records because they are over-the-counter drugs.

An indirect benefit of this laboratory strategy is that the prevalence of HB12 decreased in the post-intervention period despite the 5.9 % increase in the clinical burden. These data are generalizable to other clinical contexts, where a reduction has been achieved in the number of patients with HB12 referred to IM, despite the increase in the number of consultations. It is noteworthy that the detection of macro-B12 led to the diagnosis of 10 cases of VB12 deficiency that had remained unnoticed. Two of these cases had been previously referred to the IM consultation to study unexplained asymptomatic HB12.

The study population of the two periods shared similar characteristics. The only exception was the higher prevalence of diabetes mellitus and anemia in the pre-intervention group, a difference that the authors could not explain. A recent study demonstrated that both, VB12 elevation and deficiency are strongly associated with a higher risk for cardiovascular mortality in patients with diabetes type 2 [31]; the results of our study, however, are not consistent with that finding.

In our study cohort, the results obtained show a higher morbidity, expressed in a higher overall prevalence of neoplasm and metastasis, and mortality in the post-intervention group. The greater complexity of the subjects in the post-intervention group translated into a higher cost per patient in terms of medical consultations and imaging studies. Therefore, patient characterization improved with the laboratory intervention, thereby avoiding HB12 misdiagnosis and the associated unnecessary consultations and costs. We posited that the pre-intervention group may have included patients with HB12 which values were due to macro-B12 and, hence resulting in a lower disease burden.

There is cumulative evidence of interference from immune complexes. However, there is little literature about the impact of the laboratory’s getting involved in healthcare practice and its economic impact [5, 17]. The primary objective of this study was to assess the effect of a laboratory intervention in clinical practice. Other authors have adopted this approach in the past and used new laboratory models actively centered in decision-making [32], [33], [34], [35]. The results obtained demonstrate that this laboratory intervention was cost-effective. The laboratory invested €2,433 in equipment for the detection and elimination of interference from immune complexes. This amount is three times lower than the difference of healthcare costs generated between the pre- and post-intervention period in a single area: internal medicine. This saving might be even higher if it is generalized to the total of patients.

A limitation of this study was that the cases of HB12 managed in primary care were excluded from our estimation of the impact of the laboratory strategy on IM consultations. Nevertheless, in our health district, most cases of unexplained HB12 are referred to IM. Additionally, there is a small percentage of patients with persistent unexplained HB12, even though macro-B12 has been ruled out. In these cases, in which clinical symptoms are not consistent with laboratory results, laboratory-clinician interaction gains relevance, as it allows to find solutions to the discrepancies detected.

In conclusion, it is necessary that the laboratory plays an active role in the adequate characterization of HB12. In this context, the detection and elimination of interference from immune complexes is crucial, as it contributes to reducing the volume of diagnostic studies, optimizing decision-making and reducing costs. This study demonstrates the relevant role that the laboratory plays in clinical practice and health economics.
